# Development and Validation of a National System for Routine Monitoring of Mortality in People Recently Released from Prison

**DOI:** 10.1371/journal.pone.0157328

**Published:** 2016-06-16

**Authors:** Stuart A. Kinner, Simon J. Forsyth

**Affiliations:** 1 Griffith Criminology Institute & Menzies Health Institute Queensland, Griffith University, Brisbane, Queensland, Australia; 2 Melbourne School of Population and Global Health, University of Melbourne, Melbourne, Victoria, Australia; 3 Mater Research Institute, University of Queensland, Brisbane, Queensland, Australia; 4 School of Public Health and Preventive Medicine, Monash University, Melbourne, Victoria, Australia; 5 Centre for Adolescent Health, Murdoch Childrens Research Institute, Melbourne, Victoria, Australia; 6 School of Public Health, University of Queensland, Brisbane, Queensland, Australia; Leibniz Institute for Prevention Research and Epidemiology (BIPS), GERMANY

## Abstract

**Background:**

People released from prison are at increased risk of death. However, no country has established a system for routine monitoring of mortality in this population. The aims of this study were to (a) evaluate a system for routine monitoring of deaths after release from prison in Australia and (b) estimate the number of deaths annually within 28 and 365 days of prison release from 2000 to 2013.

**Methods:**

Persons released from prison and deaths were identified in records held by Centrelink, Australia’s national provider of unemployment benefits. Estimates generated in this manner were compared with those from a study that probabilistically linked correctional records with the National Death Index (NDI), for each calendar year 2000 to 2007. Using Centrelink data, national estimates of mortality within 28 and 365 days of release were produced for each calendar year 2000 to 2013.

**Findings:**

Compared with estimates based on linkage with the NDI, the estimated crude mortality rate based on Centrelink records was on average 52% lower for deaths within 28 days of release and 24% lower for deaths within 365 days of release. Nationally, over the period 2000 to 2013, we identified an average of 32 deaths per year within 28 days of release and 188 deaths per year within 365 days of release. The crude mortality rate for deaths within both 28 and 365 days of release increased over this time.

**Conclusions:**

Using routinely collected unemployment benefits data we detected the majority of deaths in people recently released from prison in Australia. These data may be sufficient for routine monitoring purposes and it may be possible to adopt a similar approach in other countries. Routine surveillance of mortality in ex-prisoners serves to highlight their extreme vulnerability and provides a basis for evaluating policy reforms designed to reduce preventable deaths.

## Introduction

Prisoners have complex health problems including high rates of mental illness and drug dependence.[1–3] In the context of reduced environmental risk and low-threshold, high-intensity health services, many prisoners experience marked health improvements in custody.[4] However, release from custody typically precipitates a period of acutely elevated risk of death and at least in the first year after release from custody, the vast majority of deaths are from preventable causes.[5] Among the leading causes of preventable death in this population are drug overdose, suicide, injury and, in some countries, homicide.[6–8] A recent meta-analysis of this literature showed that the risk of drug-related death remains elevated for at least four weeks after release from custody, compared with the subsequent eight weeks.[9] However, there is growing evidence that compared with demographically matched community peers, the risk of death in ex-prisoners remains significantly elevated for many years after release.[8, 10, 11]

In a number of countries including the United States, the United Kingdom and Australia,[12–14] deaths in custody are subjected to a high level of scrutiny, and are routinely monitored and publicly reported. In Australia, deaths in prison and police custody have been monitored since 1980 and the rate of death more than halved from a high of 0.44 per 100 prisoners in 1997/98 to 0.17 per 100 prisoners in 2012/13. In 2012–13 there were 53 deaths in Australian prison custody, with at least 32 of these deaths (60%) due to natural causes.[14] Although the risk of death is markedly higher after release from prison than in custody, no country routinely monitors mortality in recently released prisoners.

Using prisoner population data and crude mortality rates from two large record linkage studies, one Australian study estimated that the annual number of deaths within four weeks of release from prison was greater than the annual number of deaths in custody, and that the annual number of deaths within a year of release was as much as ten times the annual number in custody. Given the uncertainty around these estimates, the authors recommended the development of a system for routine, national monitoring of mortality in recently released prisoners, ideally through data linkage.[15]

Australia has a well-developed capacity for data linkage[16] and at least four Australian studies have used this approach to examine mortality outcomes in ex-prisoners.[8, 17–20] However, at present this data linkage infrastructure is not used for routine monitoring purposes. One alternative approach involves Centrelink, Australia’s national provider of income support payments. Because the majority of prisoners receive a one-off ‘crisis payment’ at the time of release, Centrelink data can be used to approximate the relevant denominator–that is, persons released from custody. Similarly, because many ex-prisoners remain on unemployment benefits for an extended period of time[21, 22] and death is recorded as one reason for termination of benefits, it may be possible to use Centrelink data to approximate the numerator–that is, deaths.

The aim of this study was to consider the feasibility of using Centrelink records to routinely monitor deaths in ex-prisoners in Australia, by comparing these records with those from a presumed ‘gold standard’ data linkage study of mortality in the state of Queensland. Specifically, we sought to (a) determine what proportion of releases from prison and deaths among recently released prisoners is captured in Centrelink data, (b) quantify any sampling bias in the subset of releases and deaths captured by Centrelink data, and (c) using Centrelink data, estimate the number of deaths among ex-prisoners in Australia within 28 and 365 days of release, for each calendar year 2000–2013.

## Methods

Data for the present study came from two sources: routinely collected Centrelink records, and the Mortality After Release from Custody (MARC) study–a data linkage study involving all adults released from custody in Queensland from 1994–2007.

### Centrelink data

Centrelink is a program within the Australian Government Department of Human Services that has responsibility for providing income support payments. People being released from prison often need to claim payments to meet their basic needs. If they are eligible for payments on release, have been in prison for at least 14 days and are in hardship they are also eligible for a Crisis Payment–Prison Release payment. Crisis Payment was introduced in November 1999 in recognition of the very high unemployment rate in ex-prisoners.[21, 23] In all Australian jurisdictions prisoners are routinely offered the opportunity to register for Centrelink payments prior to release. Those who are ineligible for advance registration, such as people released from court, can claim a payment on the day of release. Therefore, the record of Centrelink Prison Release payments may serve as a proxy source of information on the population of persons being released from prison. Importantly this includes both sentenced prisoners and those on remand (pre-trial detention), who in Australia constitute 27% of adults in custody.[24] Remandees may experience particularly poor post-release outcomes, partly because they are ineligible for most programs and their release is often unexpected.[25]

Centrelink also records reasons for cessation of income support payments; among the possible reasons are death and return to custody. When death is notified and verified, income support payments are cancelled. In addition to notification by family, hospitals, police and other sources, data matching is provided through a monthly linkage with the State and Territory Registrar–General’s Office to identify cases where death has not been notified. This happens on a routine basis and forms part of service provision. Cessation of benefits due to death may therefore serve as a proxy source of information on deaths in the population of ex-prisoners, although this would only ascertain those on benefits at the time of death.

We identified Centrelink records for all adults released from Australian prisons from 1 January 2000 to 31 December 2013, who claimed a Centrelink Prison Release payment. Those eligible for such payments had spent 14 days or more in custody, qualified for an income support payment and were deemed to be in financial hardship. Observation time was censored for those on income support at the date of re-incarceration or death. For the purposes of comparison with MARC data, releases from prison in Queensland during 2000–2007 were identified.

### MARC data

The MARC study involved probabilistic linkage of the identities of all adults released from full-time custody in Queensland from 1 January 1994 until 31 December 2007, with mortality data from the National Death Index (NDI). The NDI has excellent sensitivity and specificity for identifying deaths in Australia,[26, 27] including in incarcerated populations.[28] Given evidence that the inclusion of aliases increases the sensitivity of linkage without adversely affecting specificity,[29] up to 13 aliases were included in the linkage process. From a cohort of 42,015 persons released from prison, followed for a median of 7.6 years in the community, 2,329 deaths in the community were identified. The study design is described in more detail elsewhere.[5, 19, 20]

For the purposes of comparison with Centrelink data, the cohort was restricted to those released 2000–2007. Ethical clearance for the MARC study was provided by the University of Queensland’s *Behavioural and Social Sciences Ethical Review Committee*, the Queensland Corrective Services *Research Committee*, the Queensland Health *Human Research Ethics Committee* and the Australian Institute of Health and Welfare *Ethics Committee*.

### Data analysis

Within each data set and for each calendar year, we calculated the number of unique prisoners released; the number of prisoners who died within 28 days and 365 days of release; and the crude mortality rates (CMRs) within 28 days and 365 days of release.

#### Aim 1: Comparing mortality estimates

From each data set and for each calendar year 2000 to 2007, we calculated the number of persons released from custody at least once. Observation time commenced from the first release during the calendar year and was censored 365 days from the last release in that calendar year, at reincarceration (for those on unemployment benefits at the time of reincarceration) or at a release in the subsequent year, or at death.

Then, separately for each dataset and for each calendar year, we identified deaths within 28 days and 365 days of a release and computed the crude mortality rates (CMRs) per 1000 person years (py) as the number of deaths divided by cumulative years at risk, multiplied by 1000.

Using this approach we produced two indicators for each calendar year: (1) the estimated number of deaths within 28 days and 365 days of a release in that year; and (2) the estimated CMR during 28 days and 365 days from release in that year, with an associated 95% confidence interval (95%CI). For each calendar year we compared the indicators produced from MARC and Centrelink data.

#### Aim 2: Comparing the characteristics of persons released and deceased

For each data set, aggregated over years of release (2000–2007), we characterised those released, those who died within 28 days of release and those who died within 365 days of release, according to age (<25 years *vs*. 25+ years), sex (male/female) and Indigenous status (Indigenous/non-Indigenous).

#### Aim 3: Estimating national ex-prisoner mortality

Finally, using national Centrelink data for each calendar year from 2000 to 2013, we estimated the number of deaths and the CMRs within 28 days and 365 days of release, overall and according to Indigenous status, using the method described above. We estimated the relative risk (RR) and associated 95% confidence interval for the mortality trend per year, for both follow-up periods, using a general linear model.

All analyses were conducted in SAS version 9.3.[30] Mortality curves presented in Figures were smoothed using a third-order Bezier spline to accommodate non-linear trends.

## Results

For each data source and for each calendar year 2000 to 2007, Table 1 shows the estimated number of persons released from prison and the CMR within 28 days of release. Treating MARC data as the gold standard, Centrelink data under-estimated the number of persons released by a median of 5.7%, and underestimated the CMR by a median of 52.0%.

**Table 1:** Estimated number of releases and crude mortality rate within 28 days of release, among adults released from prison in Queensland 2000–2007, according to data source.

	MARC data		Centrelink data	
Year of release	Persons released	CMR per 1000 py (95%CI)	Persons released	CMR per 1000 py (95%CI)
2000	6524	16.3 (8.5–31.3)	5944	8.1 (3.1–21.7)
2001	4684	17.9 (8.5–37.6)	4433	2.7 (0.4–19.4)
2002	5059	16.6 (7.9–34.7)	4901	7.5 (2.4–23.1)
2003	4835	17.5 (8.4–36.8)	4529	8.1 (2.6–25.1)
2004	5162	28.1 (16.0–49.5)	4884	19.9 (10.0–39.8)
2005	5443	15.7 (7.5–32.9)	5167	16.5 (7.9–34.7)
2006	5692	10.6 (4.4–25.5)	5317	4.6 (1.1–18.3)
2007	6511	14.3 (7.2–28.6)	6121	11.5 (5.2–25.7)

*Note*: number of deaths according to Centrelink records has been suppressed, due to privacy requirements.

**Fig 1:**
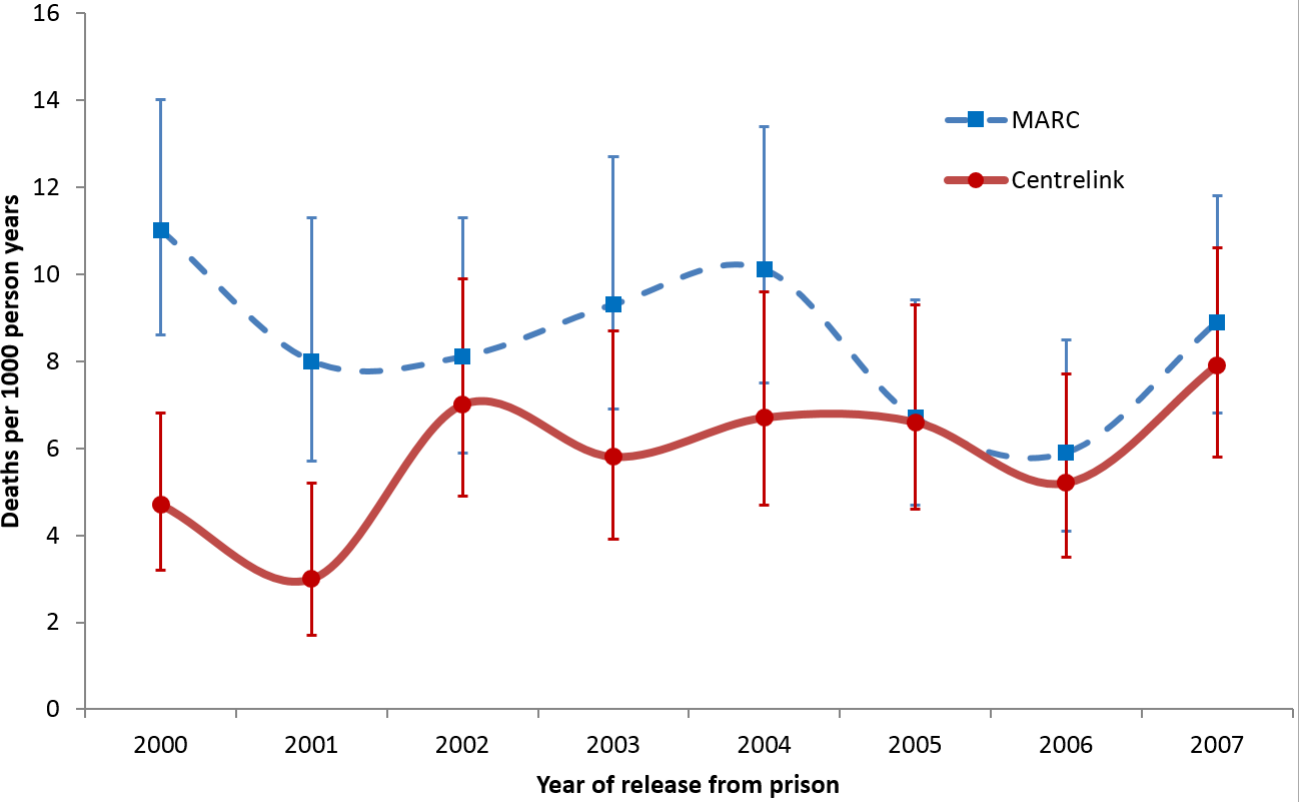
Estimated CMR within 365 days of release, based on MARC and Centrelink data.

For each data source and for each calendar year 2000 to 2007, Table 2 shows the estimated number of persons released from prison, the number of deaths and the CMR per 1000 py within 365 days of release. Treating MARC data as the gold standard, Centrelink data under-estimated the number of persons released by a median of 5.7%, person time by a median of 2.1% and the number of deaths by a median of 24.4%, although there was evidence of convergence in MARC and Centrelink estimates in later years (Fig 1). The CMR estimated using Centrelink data was on average 23.6% lower than that estimated using MARC data.

**Table 2:** Estimated number of releases, deaths and CMR within 365 days of release, among adults released from prison in Queensland 2000–2007, according to data source.

		MARC data				Centrelink data		
Year of release	Persons released	Person time at risk (years)	Deaths	CMR per 1000 py (95%CI)	Persons released	Person time at risk (years)	Deaths	CMR per 1000 py (95%CI)
2000	6524	5918.8	65	11.0 (8.6–14.0)	5944	5581.5	26	4.7 (3.2–6.8)
2001	4684	4000.9	32	8.0 (5.7–11.3)	4433	4058.8	12	3.0 (1.7–5.2)
2002	5059	4425.1	36	8.1 (5.9–11.3)	4901	4459.8	31	7.0 (4.9–9.9)
2003	4835	4278.4	40	9.3 (6.9–12.7)	4529	4128.7	24	5.8 (3.9–8.7)
2004	5162	4572.2	46	10.1 (7.5–13.4)	4884	4470.8	30	6.7 (4.7–9.6)
2005	5443	4797.2	32	6.7 (4.7–9.4)	5167	4724.9	31	6.6 (4.6–9.3)
2006	5692	4932.1	29	5.9 (4.1–8.5)	5317	4807.8	25	5.2 (3.5–7.7)
2007	6511	5589.5	50	8.9 (6.8–11.8)	6121	5473.5	43	7.9 (5.8–10.6)

Collapsed across the years 2000 to 2007, Table 3 compares the characteristics of persons released from prison and those identified as deceased, using MARC and Centrelink data. Centrelink data under-estimated the population at risk by 7.9% and substantially under-ascertained deaths within both 28 days (by 45%) and 365 days (by 33%) of release. Although the demographic characteristics of those released and deceased were similar based on MARC and Centrelink data, the crude mortality rates estimated using MARC data were significantly higher than those using Centrelink data, for deaths within both 28 days (rate ratio = 1.7) and 365 days (rate ratio = 1.5) of release (Table 3).

**Table 3:** Characteristics of releases and deaths among adults released from Queensland prisons 2000–2007, according to data source.

Characteristic	MARC data	Centrelink data
Number of releases	49018	45146
Age at release <25 (%)	15362 (31%)	14063 (31%)
Male (%)	43550 (89%)	40303 (89%)
Indigenous (%)	12737 (26%)	13420 (30%)
Deaths within 28 days	62	34
CMR (95% CI)	16.9 (13.2–21.7)	10.0 (7.1–13.9)
Age at death <25 (%)	15 (20%)	7 (21%)
Male (%)	58 (94%)	34 (100%)
Indigenous (%)	9 (15%)	4 (12%)
Deaths within 365 days	330	222
CMR (95% CI)	8.6 (7.7–9.5)	5.9 (5.2–6.7)
Age at death <25 (%)	57 (17%)	41 (18%)
Male (%)	308 (93%)	205 (92%)
Indigenous (%)	61 (19%)	52 (23%)

**Fig 2:**
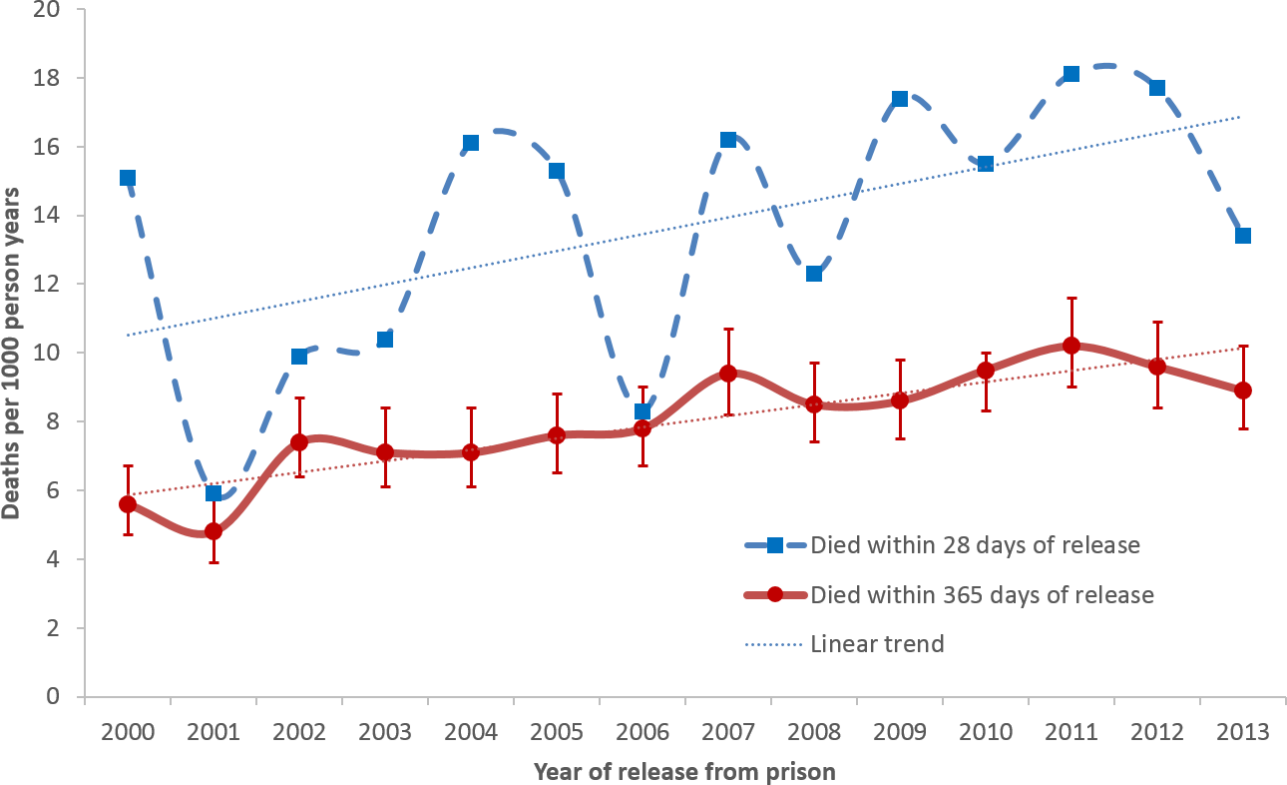
National trend of deaths after release from prison 2000–2013, based on Centrelink data.

**Fig 3:**
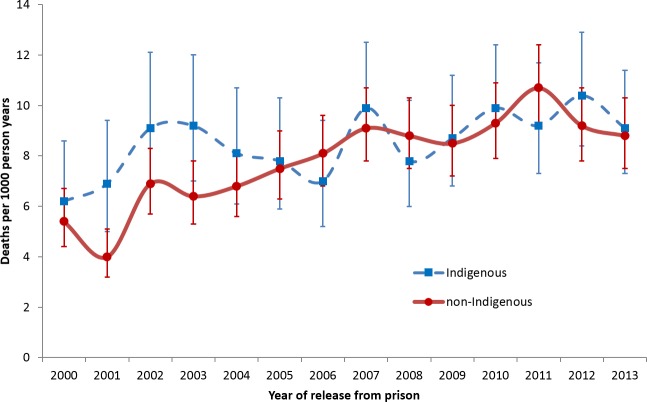
Estimated CMR within 365 days of release nationally, 2000–2013, based on Centrelink data, by Indigenous status.

Using Centrelink data, Table 4 and Fig 2 show the estimated number of persons released from prison nationally, the estimated number of deaths nationally within 28 days and 365 days of release, and the corresponding CMRs for each calendar year 2000 to 2013. The estimated CMR within 28 days of release ranged from 5.9 to 18.1 deaths per 1000 py, with a significant increase in mortality risk over time of 3.8% per year (RR = 1.038; 95%CI 1.014–1.063). The estimated CMR within 365 days of release ranged from 4.8 to 10.2 deaths per 1000 py, again with a significant increase in mortality risk of 3.4% per year (RR = 1.034; 95%CI 1.024–1.044). This increasing trend was evident for both Indigenous and non-Indigenous ex-prisoners (Fig 3). In all years, deaths occurred disproportionately in the first 28 days after release.

**Table 4:** Centrelink estimate of mortality within 28 and 365 days of release from custody in Australia, 2000–2013.

		Within 28 days of release		Within 365 days of release	
Year of release	Persons released	Deaths	CMR per 1000 py (95%CI)	Deaths	CMR per 1000 py (95%CI)
2000	24389	31	15.1 (10.6–21.5)	125	5.6 (4.7–6.7)
2001	23970	<20	5.9 (3.4–10.4)	104	4.8 (3.9–5.8)
2002	24106	20	9.9 (6.4–15.3)	161	7.4 (6.4–8.7)
2003	24047	21	10.4 (6.8–16.0)	153	7.1 (6.1–8.4)
2004	24577	33	16.1 (11.4–22.6)	154	7.1 (6.1–8.4)
2005	25601	33	15.3 (10.9–21.6)	171	7.6 (6.5–8.8)
2006	25686	<20	8.3 (5.3–13.2)	174	7.8 (6.7–9.0)
2007	27672	38	16.2 (11.8–22.2)	226	9.4 (8.2–10.7)
2008	28597	30	12.3 (8.6–17.6)	208	8.5 (7.4–9.7)
2009	28329	42	17.4 (12.8–23.5)	207	8.6 (7.5–9.8)
2010	28837	38	15.5 (11.3–21.40	233	9.5 (8.3–10.8)
2011	28806	45	18.1 (13.5–24.3)	249	10.2 (9.0–11.6)
2012	29866	46	17.7 (13.3–23.6)	239	9.6 (8.4–10.9)
2013	31189	36	13.4 (9.6–18.5)	231	8.9 (7.8–10.2)

*Note*: Cell values <20 have been suppressed for privacy reasons

## Discussion

People released from prison are at dramatically increased risk of death, particularly in the first four weeks post-release.[9] Yet despite well-established systems for routinely monitoring deaths in custody in a number of countries,[12–14] no country has yet established a system for routinely monitoring deaths after release from custody. Routine surveillance of adverse health outcomes is a critical component of a responsive, evidence-based health system.[31] In this study we examined the feasibility of using a national unemployment benefits database to routinely monitor deaths soon after release from prison in Australia. We found that these routinely collected data substantially under-ascertained mortality, particularly in the first four weeks post-release, but we found little evidence of bias in this under-ascertainment and some evidence that ascertainment of deaths using Centrelink records improved over time. Importantly, although Centrelink data did not ascertain all deaths, the results are not an estimate but rather a count of deaths among those identified as released from prison, such that the actual number of deaths among ex-prisoners in Australia each year is *at least the number* reported by Centrelink.

Over the period 2000 to 2013 Centrelink data identified an average of 32 deaths per year within 28 days of release from prison and 188 deaths per year within 365 days of release from prison. Over the same period there was an average of 47 deaths in Australian prison custody each year.[14] Given our finding that Centrelink records substantially under-estimate mortality after release from prison, it seems reasonable to conclude that the number of deaths in Australia within four weeks of release from prison is similar to the number of deaths in prison custody each year, while the number of deaths within a year of release from prison is many times greater. Our findings therefore provide compelling grounds for both concerted efforts to reduce preventable mortality in ex-prisoners, and routine monitoring of deaths in this population to enable evaluation of these efforts. Given the profound and increasing over-representation of Indigenous people in Australian prisons,[24] these efforts will be an important component of closing the gap in life expectancy for Indigenous Australians.[19]

Australia has a well-developed capacity for data linkage and this approach has been used on a number of occasions to study patterns of mortality in ex-prisoners.[5, 8, 18] An important strength of this approach is that it is likely to provide reasonably accurate estimates of mortality, however there are significant barriers to the use of data linkage for routine monitoring of mortality in ex-prisoners. First, linkage of correctional and death records is resource-intensive and requires both multiple ethics approvals and considerable in-kind support from correctional data custodians. Routine monitoring of deaths soon after release from prison may not be considered a priority by these agencies. Second, the National Death Index aggregates state-based mortality registers and given the considerable delay in collating, processing and making national mortality data available for linkage purposes, it seems unlikely that routine linkage with the NDI could be undertaken in a sufficiently timely manner to permit meaningful responses to changing trends in ex-prisoner mortality. Although these challenges are surmountable, identifying solutions will require time, money and political will–commodities that may be in short supply for such a politically and socially marginalised population. In the interim, our findings show that it is at least technically possible to routinely monitor deaths after release from prison in Australia, using a single federal government database. Critical characteristics of this database include that it identifies the population at risk, and routinely identifies deaths in this population in a timely manner. Databases with similar characteristics may be available in other countries. In some countries, it may already be possible to routinely monitor mortality after release from prison using data linkage or other means, subject to appropriate approvals and resourcing.

Our study had a number of limitations. First, although there is good evidence that probabilistic linkage with the NDI is quite accurate,[26, 27] including with prisoners,[28] it is not a true ‘gold standard’. As such, discrepancies between MARC and Centrelink may have reflected errors in our linkage with the NDI, as well as under-ascertainment of deaths in Centrelink data. Second, our validation work was based on a comparison with a cohort in Queensland, one of eight Australian jurisdictions. Although the characteristics of prisoners differ meaningfully between Australian jurisdictions,[24] we have no reason to suspect systematic differences in any under-ascertainment bias between jurisdictions. Third, although we were able to censor observation time for those who returned to custody while receiving unemployment benefits, this was not possible for ex-prisoners who were not receiving benefits at the time of reincarceration. As a consequence, we would have over-estimated person time and thus under-estimated crude mortality rates using Centrelink data. Fourth, we were unable to censor for international migration or deaths in other countries. Although migration is very rare in this population, this may have resulted in very modest over-estimation of person-time, under-ascertainment of mortality, and thus under-estimation of mortality rate. Finally, although we were able to show that the demographic characteristics of those identified through the MARC study and Centrelink records were similar, we were unable to rule out sampling bias related to other important characteristics such as substance dependence or mental disorder. Routine and timely monitoring of deaths through mechanisms such as this is not a substitute for detailed investigation of mortality in ex-prisoners through data linkage or other means.[32]

## Conclusions

People released from prison are at markedly increased risk of preventable death, particularly in the first four weeks post-release. Despite well-established systems for monitoring deaths in custody in a number of countries, there is as yet no comparable system for monitoring deaths soon after release from custody in any country. In this study we demonstrated the technical feasibility of using a national unemployment benefits database to routinely monitor deaths after release from prison in Australia. Our findings demonstrate a cost-effective method of monitoring a key health outcome for a distinctly vulnerable population. We highlight the critical importance of routinely monitoring deaths in this population, both to focus attention on the urgent need for preventive efforts, and to provide a platform for evaluation of the impact of those efforts.

## Abbreviations

MARC Study: Mortality After Release from Custody Study
NDI: National Death Index
